# Water- and Plant-Mediated Responses of Ecosystem Carbon Fluxes to Warming and Nitrogen Addition on the Songnen Grassland in Northeast China

**DOI:** 10.1371/journal.pone.0045205

**Published:** 2012-09-19

**Authors:** Li Jiang, Rui Guo, Tingcheng Zhu, Xuedun Niu, Jixun Guo, Wei Sun

**Affiliations:** 1 Key Laboratory for Vegetation Ecology, Ministry of Education, Institute of Grassland Science, Northeast Normal University, Changchun, Jilin Province, P. R. China; 2 Institute of Environment and Sustainable Development in Agriculture, Chinese Academy of Agricultural Sciences, Key Laboratory of Dryland Agriculture, Ministry of Agriculture, Beijing, P. R. China; 3 Key Laboratory of Molecular Enzymology and Engineering of Ministry of Education, College of Life Sciences, Jilin University, Changchun, Jilin Province, P. R. China; DOE Pacific Northwest National Laboratory, United States of America

## Abstract

**Background:**

Understanding how grasslands are affected by a long-term increase in temperature is crucial to predict the future impact of global climate change on terrestrial ecosystems. Additionally, it is not clear how the effects of global warming on grassland productivity are going to be altered by increased N deposition and N addition.

**Methodology/Principal Findings:**

In-situ canopy CO_2_ exchange rates were measured in a meadow steppe subjected to 4-year warming and nitrogen addition treatments. Warming treatment reduced net ecosystem CO_2_ exchange (NEE) and increased ecosystem respiration (ER); but had no significant impacts on gross ecosystem productivity (GEP). N addition increased NEE, ER and GEP. However, there were no significant interactions between N addition and warming. The variation of NEE during the four experimental years was correlated with soil water content, particularly during early spring, suggesting that water availability is a primary driver of carbon fluxes in the studied semi-arid grassland.

**Conclusion/Significance:**

Ecosystem carbon fluxes in grassland ecosystems are sensitive to warming and N addition. In the studied water-limited grassland, both warming and N addition influence ecosystem carbon fluxes by affecting water availability, which is the primary driver in many arid and semiarid ecosystems. It remains unknown to what extent the long-term N addition would affect the turn-over of soil organic matter and the C sink size of this grassland.

## Introduction

Due to rising atmospheric concentrations of CO_2_ and other greenhouse gases, global mean air temperatures increased by ≈0.2°C per decade in the past 30 years, and are projected to increase continually by 1.0 to more than 4.0°C by the end of the 21^st^ century [1], [2]. Such temperature changes, unprecedented in modern times, are predicted to substantially influence ecosystem processes and global carbon cycling [3]. Grasslands are considered to be highly relevant for future projections of the global carbon budget, as they cover approximately one-third of the earth’s terrestrial surface and store 10–30% of the world’s soil carbon [4].

Effects of warming on carbon fluxes of grassland ecosystems have been investigated to a certain extent [5]–[11]. In most of these studies, warming stimulated both gross ecosystem productivity (GEP) and ecosystem respiration (ER) with the net effects on the carbon balance depending on the temperature sensitivity of carbon release by respiration relative to carbon uptake through photosynthesis [12]. Lack of consistent pattern both across ecosystems [3] and within a grassland among growing seasons indicates that a general trend of warming on carbon balance cannot be easily predicted, as biomes exhibit a system specific and temporarily dynamic response to warming [13]. This dynamic response is due to temporal shifts in species composition and co-limitation by other abiotic resources. Dominant drivers of productivity in many grassland systems are water and nitrogen availability, both of which could strongly interact with changes in temperature [14], [15].

Many temperate ecosystems are predicted to experience rates of atmospheric N deposition as high as 2–5 g m^−2^ yr^−1^ above the preindustrial rates over this century [16]. In addition to the projected changes in temperature and water availability, increased N input by wet deposition [17] and increased grassland land-use intensity in combination with N fertilizer application are determining factors of carbon balance of grassland ecosystems. Higher N availability in grassland systems is expected to increase aboveground productivity because of enhancement in leaf area index, and improvement in ecosystem water use efficiency [18], [19]. Thus, increased N availability may, by improving water use efficiency, relieve warming-induced water deficits in semiarid grassland systems. Moreover, tightly coupled C and N cycles are strongly regulated by water availability in arid and semiarid grasslands with the combined effects of the availability of these resources ultimately determining the net impact of global change on the carbon balance of grassland systems [10], [20].

To understand the effects of global warming and changes in nitrogen availability on the carbon fluxes of grassland ecosystems, we conducted a 4-year artificial warming and N addition experiment in a meadow steppe in Northeast China. The specific questions we addressed in this study included: (1) to what extent do warming and N addition affect GEP and ER and (2) what are the interactive effects between warming and N addition on ecosystem carbon fluxes in the Songnen grassland?

## Materials and Methods

### Ethics Statement

No specific permissions were required for the described field studies, because the Songnen Grassland Ecological Research Station is a department of Northeast Normal University. No specific permissions were required for this study, because the performance of this study follows the guidelines set by Northeast Normal University. No specific permissions were required for these locations/activities. No location is privately-owned or protected in any way and the field studies did not involve endangered or protected species.

**Figure 1:**
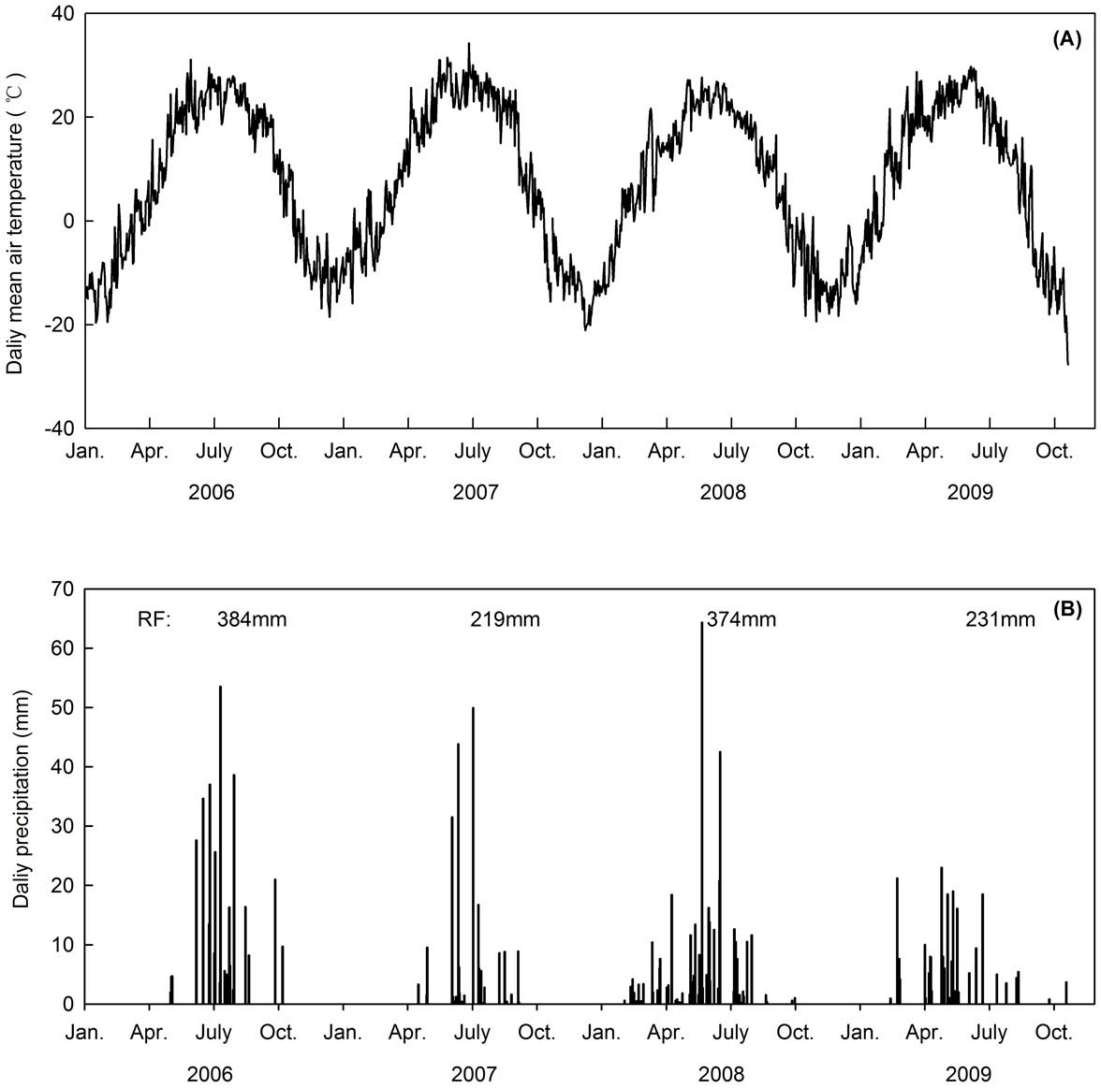
Daily mean air temperature, annual rainfall (RF), and daily precipitation in 2006, 2007, 2008 and 2009. Data for 2006 were obtained from a weather station located in the Chang Ling Horse Breeding Farm in Jilin Province, 3 km in distance from the study site. Data in 2007, 2008 and 2009 were obtained from an eddy tower roughly 200 m distant from the experimental plots.

### Study Site

The experiment was conducted at the Grassland Ecosystem Experimental Station (44°30′–44°45′N, 123°31′–123°56′E) of the Northeast Normal University in Jilin Province, China. The Songnen grassland is 30500 km^2^ in size and located at the Eastern end of the Eurasian steppe belt [21], [22]. This area has a typical meso-thermal monsoon climate with a mean annual temperature of 6.4°C, and a frost-free period of 141 days. Mean annual rainfall is 471 mm, and occurs mainly from June to August. Annual potential evapotranspiration is 2–3 times greater than the annual rainfall. The growing season was limited to late April to early October. Chernozem is the main soil type with 2.0% of soil organic carbon content, 1.4% of soil humus, 0.15% of total N, and pH >9.0 [22]. In the Songnen grassland, *Leymus chinensis* is the dominant species; *Phragmites australis*, *Chloris virgata*, *Kalimeris integrifolia*, *Carex duriuscula*, and *Artemisia mongolica* are abundance. C_3_ grasses represent approximately 90% of the total plant biomass [22], [23]. Mean annual net aboveground primary productivity at the experimental site is 300–400 g m^–2^ yr^–1^ with peak leaf area index (LAI) of up to 4 [24], [25].

**Figure 2:**
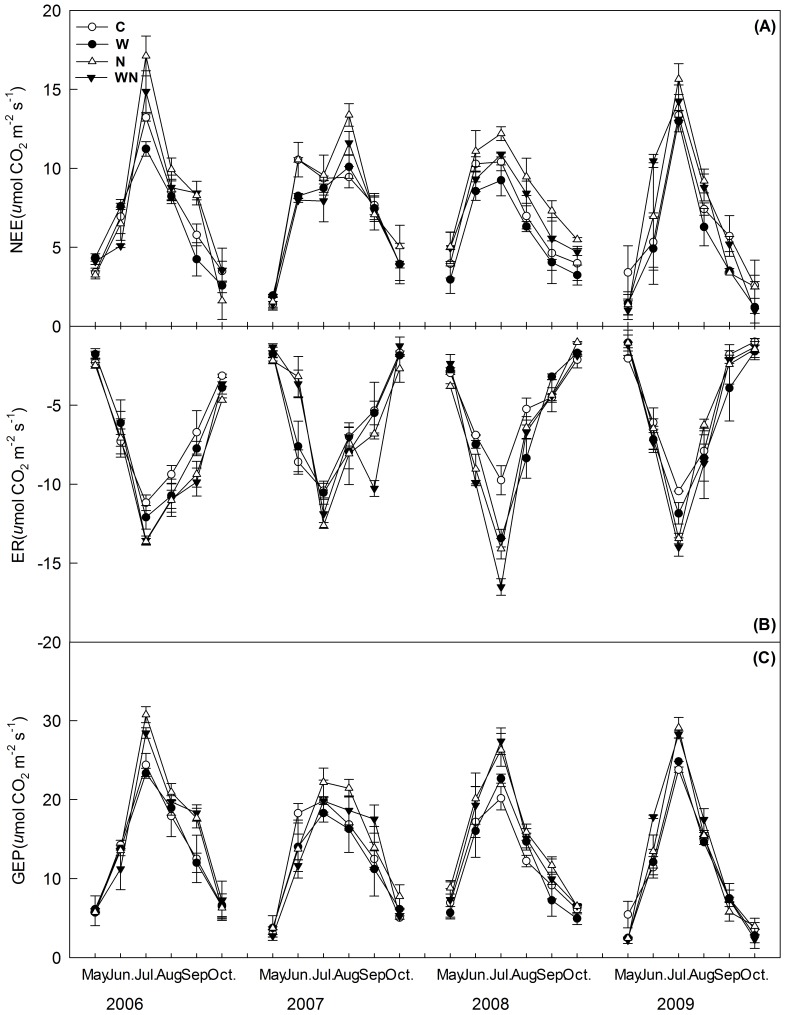
Seasonal dynamics (mean ± SE; n = 6) of net ecosystem CO_2_ exchange (NEE), ecosystem respiration (ER), gross ecosystem productivity (GEP) in response to warming (1.8°C) and N addition (10 g m^−2^ yr^−1^) in the Songnen grassland. C  =  control, W  =  warming, N  =  N addition, WN  =  combined warming and N addition.

**Figure 3:**
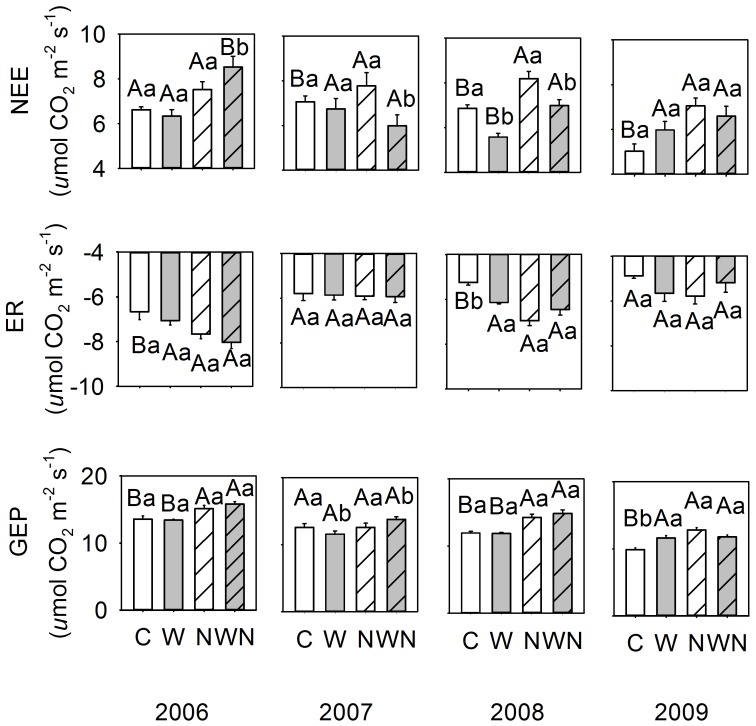
Growing season mean (n = 6) net ecosystem CO_2_ exchange (NEE), ecosystem respiration (ER), and gross ecosystem productivity (GEP). Data are presented as mean ± SE. Different letters indicate significant differences (*P*<0.05) among treatments with capital and small letters indicating differences between N and warming treatments, respectively. C  =  control, W  =  warming, N  =  N addition, WN  =  combined warming and N addition.

**Table 1:** DF, *F* values and *P* values from three-way ANOVA on the effects of warming (W), nitrogen (N), and year (Y), and their interactions on net ecosystem CO_2_ exchange (NEE), ecosystem respiration (ER) and gross ecosystem productivity (GEP).

Source	DF	NEE		ER		GEP	
		*F* Value	*P*	*F* Value	*P*	*F* Value	*P*
W	1	5.21	0.0151	5.56	0.0251	0.29	0.5935
N	1	42.53	<0.0001	14.74	0.0006	77.27	<0.0001
Y	3	8.03	0.0004	47.28	<0.0001	53.73	<0.0001
W×N	1	1.36	0.2522	0.93	0.3425	2.90	0.0990
Y×W	3	6.67	0.0014	4.22	0.0133	8.87	0.0002
Y×N	3	4.96	0.0065	1.38	0.2682	5.94	0.0026
**Y×W×N**	3	4.15	0.0143	2.89	0.0518	0.70	0.5615

**Table 2:** DF, *F* values and *P* values from repeated measures ANOVA on the effects of warming (W), nitrogen (N), and measuring date (D), and their interactions on net ecosystem CO_2_ exchange (NEE), ecosystem respiration (ER) and gross ecosystem productivity (GEP) for 2006, 2007, 2008 and 2009.

Source	DF	NEE		ER		GEP	
		*F* Value	*P*	*F* Value	*P*	*F* Value	*P*
2006							
W	1	1.111	0.323	0.662	0.44	2.124	0.183
N	1	20.964	0.002	4.712	0.062	29.271	0.001
D	5	21.606	0.002	293.997	<0.001	657.640	<0.001
W×N	1	3.680	0.091	5.572	0.046	0.001	0.979
D×W	5	4.454	0.068	1.577	0.189	3.657	0.008
D×N	5	7.644	0.024	6.321	<0.001	22.535	<0.001
D×W×N	5	114.223	<0.001	0.26	0.932	11.161	<0.001
2007							
W	1	4.843	0.059	2.804	0.133	7.765	0.024
N	1	0.001	0.973	2.289	0.163	0.462	0.516
D	5	294.033	<0.001	254.932	<0.001	469.165	<0.001
W×N	1	2.336	0.165	3.140	0.144	0.309	0.594
D×W	5	20.692	<0.001	3.783	0.007	18.103	<0.001
D×N	5	17.465	<0.001	24.125	<0.001	24.722	<0.001
D×W×N	5	8.259	<0.001	10.506	<0.001	13.290	0.005
2008							
W	1	28.119	0.001	22.054	0.002	1.078	0.329
N	1	34.156	<0.001	26.159	0.001	32.291	<0.001
D	5	153.749	<0.001	799.759	<0.001	681.520	<0.001
W×N	1	0.024	0.88	2.280	0.169	0.332	0.58
D×W	5	3.073	0.019	26.653	<0.001	7.977	<0.001
D×N	5	1.370	0.255	31.553	<0.001	10.691	<0.001
D×W×N	5	1.583	0.187	8.699	<0.001	3.126	0.018
2009							
W	1	0.433	0.529	3.106	0.116	11.058	0.01
N	1	12.162	0.008	0.250	0.63	32.985	<0.001
D	5	512.464	<0.001	688.011	<0.001	1032.304	<0.001
W×N	1	3.430	0.101	0.028	0.871	6.839	0.031
D×W	5	6.154	<0.001	2.241	0.045	6.544	<0.001
D×N	5	37.015	<0.001	13.425	0.002	35.417	<0.001
D×W×N	5	21.219	<0.001	5.396	0.014	8.739	<0.001

### Experimental Design

The experiment was carried out in a complete randomized block factorial experimental design with warming and N addition as fixed factors and each factor has two levels. The treatment combinations were un-heated and unfertilized treatment (C); unheated and fertilized treatment (N); heated and unfertilized treatment (W); and both heated and fertilized treatment (WN). Each treatment combination had 6 replications. The twelve subplots (each had an area of 3 m × 4 m) were arranged in three rows with 3 m distance between adjacent rows and 1.5 m distance between adjacent plots within each row. Warming treatment was randomly assigned to 6 of the 12 subplots; the other 6 subplots were treated as control. For each of the warming and control subplots, half of the area was treated with N addition. The airborne N deposition was estimated to be as high as 80–90 g·m^−2^·yr^−1^ and much higher N deposition is expected in the future owing to increasing anthropogenic activities and land-use change [26], [27]. For the studied temperate grassland ecosystem, the atmospheric N deposition rate was up to 2.7 g·m^−2^·yr^−1^ during the past ten years; however the saturation rate of N deposition was approximately 10.0 g·m^−2^·yr^−1^ [27], [28]. Thus, we applied 10 g·m^−2^ N in form of NH_4_NO_3_ in early June of each year in the N addition plots. Warming plots were heated continuously from June 2006 to October 2009 using MSR-2420 infrared radiators (165 cm × 15 cm; Kalglo Electronics, Bethlehem, PA, USA), suspended 2.25 m above the ground. Infrared radiators supplied 1600 W·m^−2^ of thermal radiation, resulting in a soil-surface warming of 1.7±0.2°C. The warming effects of infrared radiators were tested by measuring soil surface temperature at 20 points diagonally arranged across the heated plots in June of 2007 and 2008. Warmed plots had significantly (*P* = 0.003) higher temperature during the experimental period. In unheated control plots, ‘dummy’ heaters with the same shape and size as the infrared radiator were suspended 2.25 m above soil surface in order to simulate the shading effects of the heater.

**Table 3:** Net photosynthetic rate (*P*n), stomatal conductance (*G*s) and relative coverage (mean ± SE) of *Leymus chinensis* and *Phragmites australis* measured in June of 2006, 2007, 2008 and 2009.

		*Leymus chinensis*				*Phragmites australis*			
		2006	2007	2008	2009	2006	2007	2008	2009
	C	10.5±0.44 Aa	7.97±0.63 Aa	15.9±0.96 Aa	17.13±0.37 Aa	13.39±1.41 Aa	16.86±1.48 Aa	22.25±1.64 Aa	24.63±1.57 Aa
*P*n (*µ*molCO_2_m^−2^s^−1^)	W	9.58±0.60 Ab	8.53±1.31 Aa	16.4±0.96 Aa	17.72±2.21 Aa	15.59±0.74 Aa	14.96±1.62 Aa	19.36±0.98 Aa	27.43±0.8 Aa
	N	10.9±0.43 Aa	9.22±1.02 Aa	17.37±0.83 Aa	18.15±0.75 Aa	17.03±1.3 Aa	14.71±1.44 Aa	17.63±0.44 Ba	28.12±0.34 Aa
	WN	7.97±0.31Bb	8.79±1.08 Aa	17.42±1.15 Aa	16.72±0.8 Aa	16.61±0.77 Aa	20.23±2.56 Ba	19.78±0.66 Aa	24.03±1.72 Ab
	C	0.45±0.04 Aa	0.15±0.02 Aa	0.38±0.05 Aa	0.11±0.01 Aa	0.47±0.03 Aa	0.33±0.04 Aa	0.67±0.12 Aa	0.41±0.08 Aa
*G*s (*m*molm^−2^s^−1^)	W	0.38±0.02 Aa	0.14±0.01 Aa	0.40±0.04 Aa	0.15±0.0 Aa	0.53±0.04 Aa	0.31±0.03 Bb	0.48±0.07 Ab	0.40±0.02 Aa
	N	0.46±0.04 Aa	0.15±0.20 Aa	0.35±0.04 Aa	0.16±0.01Ba	0.55±0.06 Aa	0.303±0.03 Aa	0.55±0.03 Ba	0.54±0.03 Ba
	WN	0.35±0.03 Aa	0.17±0.16 Bb	0.46±0.04 Ab	0.16±0.02 Aa	055±0.03 Aa	0.40±0.04 Bb	0.58±0.05 Ba	0.34±0.04 Bb
	C	92±2 Aa	83±3 Aa	80±3 Aa	80±2 Aa	3±2 Aa	10±1 Aa	4±1 Aa	13±1 Aa
Relativecoverage (%)	W	91±2 Aa	82±3 Aa	71±2 Aa	75±2 Ab	5±2 Aa	13±1 Aa	3±1 Aa	10±2 Ab
	N	93±1 Aa	90±4 Ba	89±3 Ba	85±3 Ba	2±1 Aa	5±1 Ba	4±2 Aa	11±2 Ba
	WN	93±1 Aa	82±2 Ab	90±1 Bb	82±1 Bb	3±1 Aa	13±1 Aa	5±1 Aa	13±2 Bb

Measurements were performed on the upper most fully expanded leaves from 9.00 to 11.00 a.m. Different letters indicate significant differences (*P*<0.05) among treatments with capital and small letters indicating differences between N and warming treatments, respectively. C  =  control; W  =  warming; N  =  N addition; WN  =  combined warming and N addition.

### Climate Data, Soil Temperature and Soil Water Content

Climate data in 2006 were obtained from the Pasture Ecology Research Station of the Northeast Normal University, Jilin Province, China (123°44′ E, 44°40′ N), approximately 3 km distant from the study site. Data in 2007, 2008 and 2009 were obtained from an eddy tower roughly 200 m distant from the experimental plots. Soil moisture sensors and soil temperature probes were installed in the middle of each of the 24 plots to measure soil temperature at 10 cm depth and volumetric soil water content (SWC) at 0–10 cm soil depth, respectively. Soil temperature and SWC were continuously recorded at 1-h interval from June 2006 to October 2009 and data were recorded with an ECH_2_O dielectric aquameter (EM50/R Decagon Ltd, Pullman, WA, USA) from June 2006 to October 2009.

**Figure 4:**
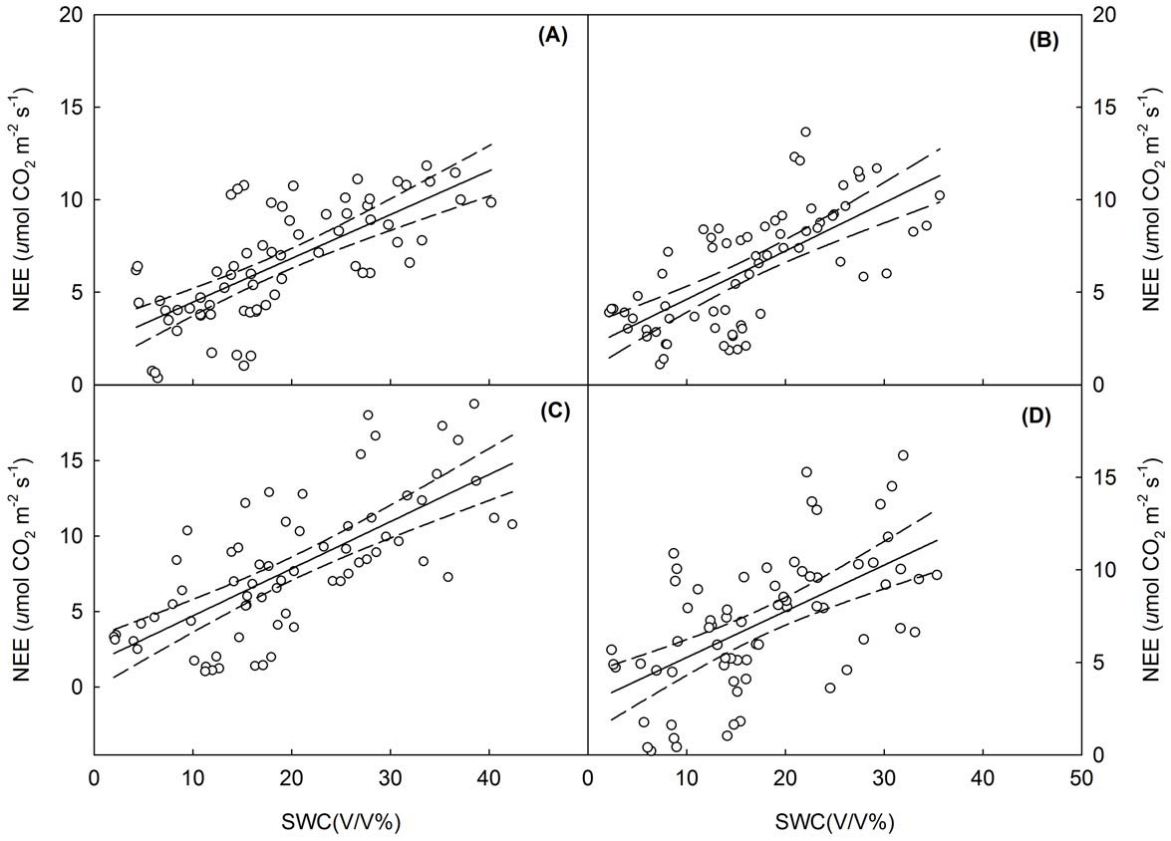
Temporal dependence of net ecosystem CO_2_ exchange (NEE) on soil water content (SWC) across the four growing seasons. Regression lines (solid) and 95% confidence level (dotted lines) are showed. All the linear regressions are statistically significant (*P*<0.05). Regression equations and significance levels are provided in Table 4. (A), control; (B), warming; (C), N addition; (D), combined warming and N addition.

**Table 4:** Summary of linear regressions describing the relationship between carbon flux component and soil water content (SWC), leaf area index (LAI) and the relationship between ecosystem respiration (ER) and gross ecosystem productivity (GEP).

		Treatment			
		Control	Warming	Nitrogen	Warming×Nitrogen
NEE(*u*molCO_2_m^−2^s^−1^) vs.SWC(V/V%)	y-intercept	2.066	2.0044	1.594	2.7729
	slope	0.2365	0.261	0.3119	0.2496
	r^2^	0.4886	0.4659	0.4972	0.3402
	MS	314.90	316.75	689.63	313.74
	*P*	<0.0001	<0.0001	<0.0001	<0.0001
ER(*u*molCO_2_m^−2^s^−1^) vs.SWC(V/V%)	y-intercept	0.9146	0.6552	0.4292	−0.2314
	slope	0.2589	0.3416	0.3001	0.3913
	r^2^	0.5177	0.5662	0.5326	0.5317
	MS	377.32	542.41	638.37	771.08
	*P*	<0.0001	<0.0001	<0.0001	<0.0001
GEP(*u*molCO_2_m^−2^s^−1^) vs.SWC(V/V%)	y-intercept	3.0095	2.6596	2.0193	2.6494
	slope	0.4954	0.6026	0.6120	0.6364
	r^2^	0.5426	0.5815	0.5781	0.5000
	MS	1381.62	1688.15	2654.78	2039.59
	*P*	<0.0001	<0.0001	<0.0001	<0.0001
NEE(*u*molCO_2_m^−2^s^−1^) vs.LAI	y-intercept	0.5412	−0.2915	−4.2295	−1.3917
	slope	4.2316	4.6648	6.3741	4.4301
	r^2^	0.2792	0.5243	0.8201	0.7253
	MS	61.19	80.9279	264.93	42.2482
	*P*	0.0242	0.0007	<0.0001	<0.0001
ER(*u*molCO_2_m^−2^s^−1^) vs.LAI	y-intercept	−0.4930	0.4373	−1.6812	−1.4304
	slope	4.2868	3.3815	3.4772	3.7369
	r^2^	0.5325	0.3872	0.6059	0.5844
	MS	62.7906	42.5253	78.84	115.25
	*P*	0.0006	0.0058	0.0001	0.0002
GEP(*u*molCO_2_m^−2^s^−1^) vs.LAI	y-intercept	0.0482	0.1458	−5.9107	−2.8220
	slope	8.5184	8.0463	9.8513	8.1670
	r^2^	0.4024	0.5054	0.8241	0.6932
	MS	247.94	240.78	632.82	550.50
	*P*	0.0047	0.0009	0.0001	<0.0001
ER(*u*molCO_2_m^−2^s^−1^) vs.GEP(*u*molCO_2_m^−2^s^−1^)	y-intercept	−0.5914	−0.6659	−0.4017	−1.2563
	slope	0.5170	0.5503	0.4793	0.5700
	r^2^	0.9351	0.9119	0.8852	0.9108
	MS	713.26	902.39	1101.40	1383.80
	*P*	<0.0001	<0.0001	<0.0001	<0.0001

### Leaf Area Index

Leaf area index (LAI) was measured monthly with a plant canopy analyzer (LAI-2000 Plant Canopy Analyzer, Li-Cor, Lincoln, NE, USA).

**Figure 5:**
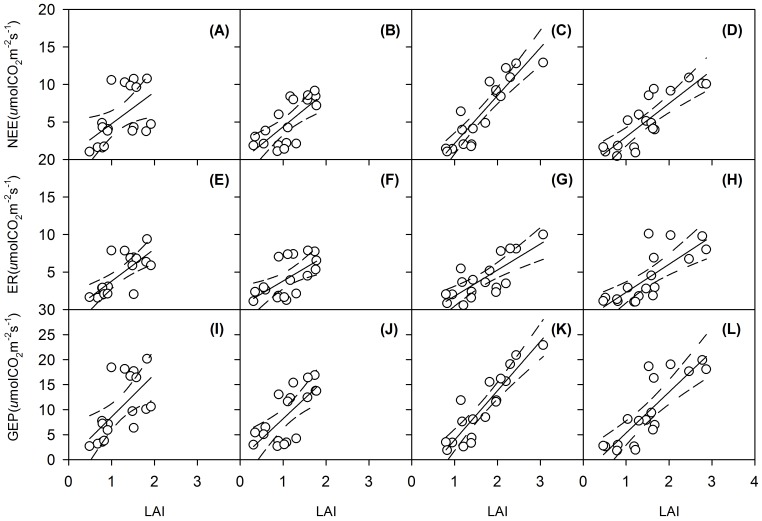
Temporal dependence of seasonal mean net ecosystem CO_2_ exchange (NEE), ecosystem respiration (ER) and gross ecosystem productivity (GEP) on leaf area index (LAI) across the four treatments in May and June of 2006–2009. Regression lines (solid) and 95% confidence level (dotted lines) are showed. All the linear regressions are statistically significant (*P*<0.05). Regression equations and significance levels are provided in Table 4. Control plots (A,E,I); Warming plots (B,F,J); N addition plots (C,G,K); Warming and N addition plots (D,H,L).

### Measurements of Ecosystem Gas Exchange

Ecosystem gas exchange was measured once a month over the growing seasons (from May to October) from 2006 to 2009. Measurements were performed under cloud-free conditions from 9 a.m. to 1 p.m. To avoid potential effects of temporal variation in CO_2_ exchange between ecosystem and the atmosphere during the 4-hour measuring period, we measured the 6 treatment combinations sequentially. In May 2006, a square aluminum frames (0.5 m × 0.5 m) were inserted into the soil to a depth of approximately 3 cm on each plot with a distance of 30 cm to the plot’s border. Care was taken to minimize soil disturbance during installation. Frames provided a plane base for the mounting of the mobile canopy chamber (0.5 m × 0.5 m × 0.9 m, Polymethyl Methacrylate).

Ecosystem gas exchange (CO_2_ and water fluxes) was measured with a closed-flow infrared gas analyzer (LI-6400, Li-Cor, Lincoln, NE, USA) attached to the transparent canopy chamber. During measurements, the chamber was sealed to the soil surface by attaching it to the permanently fixed aluminum base in each plot. Four small fans ran continuously to mix the air inside the chamber during the measurement period. Nine consecutive recordings of CO_2_ concentrations were taken at 10-s intervals during a 90-s period after steady-state conditions were achieved within the chamber (approximately 30 seconds after the placement of chamber). Net ecosystem CO_2_ exchange (NEE) was determined from the time-courses of chamber CO_2_ concentration change. We followed the approach of Steduto et al. [29] for the conversion from concentration change to flux per unit soil surface area. The NEE data are presented in the notation commonly used by the terrestrial sciences community whereby positive values represent net fluxes from the atmosphere to the ecosystem (carbon uptake) and negative values represent from the terrestrial ecosystem to the atmosphere (carbon release). Details on these static-chamber flux calculations can be found in the soil-flux calculation procedure on Page 6-2 in the LI-6400 manual (Li-Cor Inc., 2004). Following the NEE measurements, the chamber was vented, replaced on the aluminum frame, and covered with an opaque cloth to measure ecosystem respiration (ER). The ER values were obtained by monitoring steadily increasing in chamber CO_2_ concentration (usually after 1 min). Gross ecosystem productivity (GEP) was calculated as the difference between NEE and ER.

### Leaf Gas-exchange Measurements

Leaf net photosynthetic rate and stomatal conductance in *L. chinensis* and *P. australis* were measured with a Li-6400 gas analyzer (Li-Cor, Lincoln, NE, USA) between 9–11 a.m. on a monthly basis during 2006–2009. The upper most fully expanded leaves were used for leaf gas exchange measurements. Gas exchange parameters were recorded at light intensities of 1400–1600 µmol m^–2^ s^–1^ after readings stabilized (approximately 5 min). In order to compare chamber measurements of GEP with leaf gas exchange data, results from the June measurements are reported in this study.

### Statistical Analysis

Seasonal mean values used in this study were calculated from the monthly mean values, which were first averaged from all measurements in the same month. Three-way ANOVA was used to examine the effects of year, warming, N addition, and their possible interactions on ecosystem C fluxes. If significant inter-annual variation was detected (year effect *P*<0.05), Repeated Measures ANOVA (RMANOVA) were used to examine the temporal (inter- or intra-annual) variation and the effects of warming and N addition effects on ecosystem C fluxes over the growing seasons in 2006, 2007, 2008 and 2009, respectively. Between-subject effects were evaluated as warming, N addition and their interactions, and within-subject effects were year (or measuring time within season) variation and the effects of warming and N addition on ecosystem C fluxes. Bonferroni correction was used to adjust for multiple comparisons. Regression with correction for autocorrelation and stepwise multiple linear analyses were used to examine the relationships of ecosystem C fluxes with soil moisture, and leaf area index. All statistical analyses were conducted with SAS software (SAS Institute Inc., Cary, NC, USA).

## Results

### Soil Microclimate

For the four experimental years, annual mean air temperatures were higher and annual precipitation amounts were lower compared to 30-year (1980–2010) averages of 6.4°C and 471 mm (Fig. 1A, B). The manipulated warming significantly elevated soil temperature (*P* = 0.045). Throughout the whole experimental period, mean soil temperature at 10-cm depth in the heated plots was 2.34°C higher compared to that of the control plots. However, the warming effects were less pronounced in the N addition plots, with only 1.31°C increase in 10-cm soil temperature.

### Seasonal Variation in Ecosystem C Fluxes

Temporal dynamics of net ecosystem CO_2_ exchange (NEE), ecosystem respiration (ER), and gross ecosystem productivity (GEP) during the vegetation growth period followed the seasonal patterns of air temperature in the four experimental years with higher values of CO_2_ exchange being observed in summer and lower values in both spring and autumn. Comparing the seasonal dynamics of NEE of the four years, peak values were found in July of 2006 and 2009, whereas no such clear peaks were observed in the other two years. ER and GEP were always highest in July (Fig. 1 and 2).

### Warming Effects on Ecosystem C Fluxes

Pooling the data from the four experimental years together, we observed that warming had significant effects on NEE and ER, but not on GEP (Table 1). As indicated by the significant interaction between years and treatment factors (Table 1; *P* = 0.0143), warming effects on growing season NEE were not consistent over the 4 experimental years (Table 2). On unfertilized plots, warming decreased NEE in three of the four experimental years, but not in 2009 (Fig. 2A and 3; Table 2). Average growing season ER and GEP were interactively affected by warming and year (ER, *P* = 0.0133; GEP, *P* = 0.0002; Table 1). Warming increased ER in 2008 and 2009 whereas had no apparent effects in 2006 and 2007 (Fig. 2B and 3; Table 2). Warming decreased GEP in both 2007 and 2009; whereas it had no effects on GEP in 2006 and 2008 (Fig. 2C and 3; Table 2).

No effects of warming on *L. chinensis* relative coverage were observed in years of either 2006 or 2007 (Table 3). Warming marginally reduced *L. chinensis* relative coverage in years of 2008 and 2009 (11.25%, *P* = 0.053; 6.25%, *P* = 0.069; Table 3).

### Nitrogen Effects on Ecosystem C Fluxes

Compared to the control plots, NEE in the N addition and un-heated plots increased slightly in 2006, but to a large extent in 2007, 2008 and 2009 (Fig. 2A). The enhancement of N addition on ER was consistent throughout the growing seasons. N addition increased GEP in all experimental years except in 2007 with the most pronounced enhancement, up to 21%, observed in 2008. N addition marginally increased *L. chinensis* relative coverage by 8.4% (*P* = 0.063), 11.25% (*P* = 0.048), and 6.25% (*P* = 0.054) in 2007, 2008 and 2009, but had no effects in 2006 (Table 3).

### Interactive Effects of Warming and N Addition on Ecosystem C Fluxes

No interactive effects of warming plus N addition on NEE, ER, and GEP were detected when we pooled the data from the four experimental years together (Table 1). In 2006, warming plus N addition significantly increased ER, and warming plus N addition significantly increased GEP in 2009 (Table 2).

### Leaf-level Gas Exchange

For *L. chinensis*, the significant warming effects on leaf photosynthetic rate (*P*_n_) were observed in 2006, but there were no significant effects in 2007, 2008 and 2009 (Table 3). In contrast, significant N addition stimulation on stomatal conductance (*G*_s_) of *L. chinensis* was detected only in 2009 (Table 3). The *P*_n_ of *P. australis* was 20.8% lower in the N addition than in the control treatment in 2008, but had no significant effects on leaf-level gas exchange in 2006, 2007 and 2009. The *G*_s_ values of *P. australis* were 6.1 and 28.4% lower in the warming than in the control treatment in 2007 and 2008, but there are no significant effects in 2006 and 2009 (Table 3). In contrast, N addition significantly decreased *G*_s_ of *P. australis* in 2008, but increased *G*_s_ of *P. australis* in 2009 (Table 3).

### Relationships between C Fluxes, Leaf Area Index and Soil Water Content

SWC at 0–10 cm depth fluctuated greatly over the growing season. The warming treatment decreased seasonal mean SWC by 12.9% in 2006, 7.4% in 2007, 11.4% in 2008, and 25.0% in 2009 (*P*<0.05). Conversely, the nitrogen addition increased seasonal mean SWC by 10.3, 8.7, 0.5 and 10.4% in 2006, 2007, 2008 and 2009, respectively. There were no significant relationship between NEE and soil temperature (data not shown); whereas variation in NEE during the growing seasons was strongly correlated with difference in the monthly mean values of SWC across the treatments (Fig. 4; Table 4).

We observed strong positive correlations between GEP and LAI during May and June from 2006 to 2009 for the four treatments (Fig. 3 and 5; Table 4). The slopes of these linear regression functions were of similar magnitude among all treatments, ranging from 7.1 to 9.9 µmol CO_2_ (m_Leaf_)^−2^ s^−1^.

## Discussion

### Effects of Warming on Ecosystem C Fluxes

Warming can increase NEE by increasing GEP relative to ER. This, by definition, should be expected in temperature-limited environments and/or growth phases. Conversely, warming could decrease NEE if it caused a greater increase in ER than GEP. Warming induced reduction in NEE occurs when air temperature is beyond the ecosystem’s optimum temperature for carbon assimilation and/or ER responds more strongly to temperature increase than GEP. Moreover, warming could indirectively influence NEE through altering community composition [30], [31], and the availability of both water and nutrients [5], [8], [32]. Previous studies suggest that steppe is subject to a highly variable control of NEE by temperature (during the transition from winter to spring and during the carbon and nitrogen re-allocation phase at the end of the growing season), water (with transient or permanent water deficits most strongly impacting on carbon gain during the main growth period, May to August), and a co-limitation of productivity by N availability in wet years [19], [33], [34].

In this experiment on the Songnen grassland, which is subject to intensive land-use change [35], warming significantly decreased NEE and increased ER, but had no apparent effects on GEP (Table 1) when we pooled 4 years data. The effects of warming on ecosystem carbon fluxes were not consistent through the experimental period (Fig. 3 and Table 2). For instance, in 2008 the negative effects of warming on NEE could be explained by a warming-induced increase of ER with GEP remaining unaffected; whereas in 2009, warming increased NEE due to a proportionally greater increase of GEP than ER. Interpretation of these results is further complicated by interactions between warming and N addition with partially opposite effects of warming on fertilized and un-fertilized plots.

In the studies of Niu et al. [10] and Wan et al. [36] negative warming effects on NEE were predominantly of indirect nature, as changes in NEE were largely attributed to lower soil moisture. Directly supporting this finding, variation in NEE in the four experimental years coincided with changes in average monthly SWC (Fig. 4). Low SWC constrained plant growth and ecosystem productivity particularly during early spring (May). The observed reduction of CO_2_ fluxes on the same grassland in 2007 by Dong et al. [37] was also related to early spring drought; these findings together illustrate that warming alters ecosystem carbon fluxes through affecting water availability, which is one the primary drivers of carbon fluxes in this semi-arid grassland.

In the two of the 4 experimental years, we observed significant enhancement in ER in warming plots (Fig. 2 and 3; Table 2). This finding is contrary to findings of Niu et al. [10] and Xia et al. [38] who found no or only slight effects of warming on ER. Similarly, our results don’t support the finding of Xia et al. [38] that GEP was more sensitive to inter-annual climatic variation than ER. This finding implies that both carbon fluxes in our study were functionally closely linked and that both exhibited apparently similar sensitivity to intra- and inter-annual variation in climatic conditions. The results of this study, along with those performed in other biomes [39], suggest ER apparently is not independent of the carbon gain and functionality of grassland in general.

### Response of C Fluxes to N Addition

Our study focused on the analysis of interactive effects of N fertilizer application and warming on the carbon budget of the Songnen grassland during the vegetation growth periods. N addition stimulated NEE in three of the four years (Fig. 2A; Table 1), largely due to N-induced increases in GEP. N stimulation of ecosystem C fluxes in the studied grassland is in accordance with positive N responses of grassland productivity in other ecosystems [38], [40]–[42].

N stimulation of ecosystem NEE could have resulted from higher canopy photosynthesis rates. However, the slope of the linear regression between GEP and LAI indicates that there were no significant differences in canopy photosynthesis rates among the four treatments (Fig. 5). Leaf net assimilation rates were, as well, not affected by treatments (Table 3). The overall higher biomass accumulation and NEE in N addition plots were reflected in an increases in ER (Fig. 3). Enhanced aboveground activity apparently resulted in stimulation of belowground C input, and subsequent root and microbial activities. It remains unknown to what extent the long-term application of N would affect the turn-over of soil organic matter and the C sink size in the studied grassland.

Although there were no significant interactive effects of warming and N addition on ecosystem C-exchange over the experimental period (Table 1), the observed greater rates of NEE, ER and GEP in the WN plots, relative to the W plots (Fig. 5B and C), suggests that warming produces, to some extent, positive effects on ecosystem C-exchange when combined with N addition. We therefore speculate that, given enough time, there may be a significant interactive effects between warming and N addition on ecosystem carbon fluxes because of potential impacts of warming plus N addition on the dynamics of litter and soil organic C.

### Conclusions

NEE in the Songnen grassland in Northeast China was significantly affected by warming and N addition treatments, however there were no interactive effects between warming and N addition. The observed inter-annual variation in the effects of warming and N addition treatments suggests that water availability is a key driver of ecosystem carbon fluxes in the studied grassland. Contrary to common expectations of stimulated ecosystem C-fluxes in warming plots, we found reductions in all the measured C-fluxes on the Songnen grassland in northeastern China. The observed decrease in ecosystem C-fluxes could be attributed to offsetting of the direct and positive effects of elevated temperature by the indirect and negative effects via exacerbating water stress. These results highlight the uniqueness of the grassland in the semi-arid and salinate region of northeastern China in response to the enhanced temperature and nutrition levels. Increased N not only enhanced ecosystem C-fluxes, but also ameliorated the negative C balance caused by experimental warming on ecosystem C-fluxes.

## Supporting Information

Figure S1 **Seasonal development of leaf area index (LAI, mean ± SE) of the four treatments during the vegetation growth period (May to October).** C  =  control, W  =  warming, N  =  N addition, WN  =  combined warming and N addition.
(TIF)
