# Supplementary figures and images for: Water- and Plant-Mediated Responses of Ecosystem Carbon Fluxes to Warming and Nitrogen Addition on the Songnen Grassland in Northeast China
Source: PLoS One. 2012 Sep 19;7(9):e45205. doi: 10.1371/journal.pone.0045205 (PMC3446953; doi:10.1371/journal.pone.0045205)

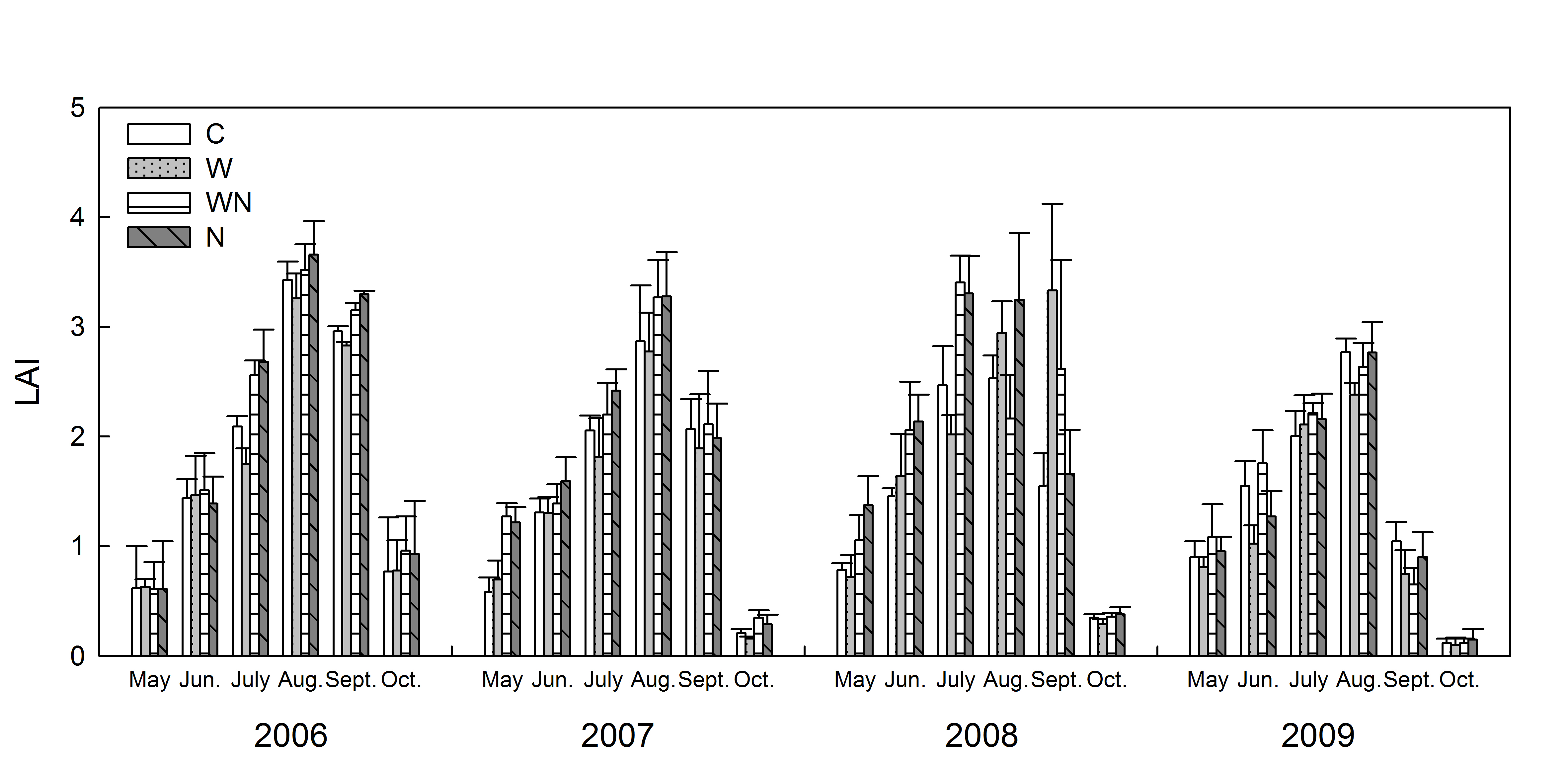

Supplement: Figure S1 — Seasonal development of leaf area index (LAI, mean ± SE) of the four treatments during the vegetation growth period (May to October). C = control, W = warming, N = N addition, WN = combined warming and N addition. (TIF) [file pone.0045205.s001.tif]
